# Acceptability, use and safety of the BlueIce self-harm prevention app: qualitative findings from the Beating Adolescent Self-Harm (BASH) randomised controlled trial

**DOI:** 10.1136/bmjment-2023-300961

**Published:** 2024-06-25

**Authors:** Paul Stallard, Kathryn Whittle, Emma Moore, Antonieta Medina-Lara, Nia Morrish, Shelley Rhodes, Gordon Taylor, Bethany Cliffe

**Affiliations:** 1 Department for Health, University of Bath, Bath, UK; 2 Oxford Health NHS Foundation Trust, Oxford, UK; 3 Department of Public Health and Sport Sciences, University of Exeter, Exeter, UK; 4 University of Exeter Medical School, Exeter, UK

**Keywords:** Child & adolescent psychiatry, Suicide & self-harm

## Abstract

**Background:**

Little is known about the social validity of self-harm prevention apps for young adolescents with severe mental health problems who repeatedly self-harm.

**Objective:**

We assessed the acceptability, use and safety of BlueIce, a self-harm prevention app for young adolescents who self-harm.

**Methods:**

Mixed methods study involving a content analysis of postuse interviews. Participants were a clinical group of 60 UK adolescents aged 12–17 with repeated self-harm, randomised to receive BlueIce.

**Findings:**

BlueIce was used by 57/60 (95%) respondents with 47/57 (82%) using BlueIce when thinking about self-harm. 17/47 (36%) who were thinking about self-harm used it on more than six occasions with 36/47 (77%) reporting that BlueIce prevented at least one episode of self-harm. 33/47 (70%) reported occasions when they used the app but still went on to self-harm. Reasons why the app was not used or not helpful included feeling too distressed, a negative mindset, prior decision to self-harm or forgetting. BlueIce was rated 4.09 (SD=0.75) out of 5 stars, with high mean ratings out of 10 for ease of use (8.70, SD=1.37) and good for acceptability (7.68, SD=2.05) and helpfulness (6.77, SD=1.72). No respondent identified BlueIce as triggering any episode of self-harm.

**Conclusion:**

These findings are consistent with previous evaluations and highlight the acceptability, use and safety of BlueIce. Self-reports indicate that BlueIce prevented some episodes of self-harm.

**Clinical implications:**

Our results highlight the acceptability of the BlueIce self-harm app for young adolescents who repeatedly self-harm.

**WHAT IS ALREADY KNOWN ON THIS TOPIC**

- Around one in four children and young people under the age of 18 will self-harm.
- Most self-harm occurs in private, peaking in the hours around midnight and rarely results in presentation to health services.
- Although digital technology offers the potential to provide accessible and immediate support, little is known about the acceptability of self-harm prevention apps for young adolescents.

**WHAT THIS STUDY ADDS**

- Feedback from 60 young adolescents aged 12–17 years who were randomised to receive a self-harm prevention app (BlueIce) was analysed.
- BlueIce was used by 95% of respondents, with 82% using the app when thinking about self-harm. Although three-quarters reported that BlueIce prevented at least one episode of self-harm, 70% reported occasions when they used BlueIce but went on to self-harm.
- BlueIce was acceptable, used and safe.

**HOW THIS STUDY MIGHT AFFECT RESEARCH, PRACTICE OR POLICY**

- The addition of BlueIce to the usual mental healthcare for young people who repeatedly self-harm could prevent some episodes of self-harm.

## Background

Community surveys indicate that up to a quarter of children will self-harm by the age of 19.^1 2^ Adolescence is the critical period for self-harm,^3^ peaking between the ages of 13 and 16 and declining after 18 years of age.^4 5^ The prevalence of self-harm during childhood has risen over the past 20 years^6^ with rates being adversely increased by COVID, particularly for girls.^7–9^ Nearly all self-harm occurs in private, peaking in the hours around midnight, with a minority of episodes resulting in health service presentations.^10–12^ Possible reasons for low rates of help seeking include personal guilt and shame, perceived stigma, fear of a negative response and difficulties accessing services.^13^

Evidence for the effectiveness of psychological interventions for children and adolescents who self-harm is limited.^14 15^ Although self-harm interventions appear acceptable, randomised trials are limited, of poor quality and have a high risk of bias.^14 15^ Dialectical Behaviour Therapy for Adolescents has the strongest evidence base which, alongside cognitive–behavioural therapy, has been identified as warranting further evaluation.^14^ Treatment components of successful studies include both children and carers, emphasise the therapeutic alliance, individualise treatment, focus on safety planning and develop emotional regulation skills.^16^

Interest in the use of digital technologies, particularly mental health apps, has been stimulated by the growing demand for, and the limited capacity of, traditional mental health services.^17 18^ Most apps can be directly accessed without professional referral, thereby reducing the potential stigma associated with help seeking. Apps offer an alternative to a face-to-face intervention which some might find embarrassing or uncomfortable, particularly when discussing self-harm.^19^ They provide timely access to mental health support and can be used where and when help is required, 24/7, outside of traditional health settings and hours. Apps often develop skills and provide opportunities to enhance acquisition through use and practice in everyday environments.^19^ They are often appealing to adolescents who are familiar with, and frequent users of, technology^18^ while providing low-cost alternatives, or supplements to, face-to-face interventions.^17^

While offering potential benefits, concerns have also been identified. First, the majority of mental health apps have been developed by technology companies with minimal clinical input^20^ resulting in app content failing to reflect evidence-based guidelines and, in some instances, potentially encouraging risky behaviours.^21^ Second, concerns about digital data privacy and unauthorised access to mental health app data are common.^22^ These concerns appear to reflect current practice where 39% of apps offered by college counselling centres had no privacy policy, and of those that did, 49% explicitly stated that they shared users’ data with third parties.^23^ Third, apps addressing self-harm and suicidal behaviour raise particular concerns about personal safety and how crises are managed. An analysis of 34 digital interventions for self-harm found that only three (9%) included a safety plan^24^ raising concerns about their responsiveness to emergencies and sensitivity to early warning signals.^25^ However, perhaps the largest concern relates to the lack of evidence to demonstrate the effectiveness of mental health apps. A recent review of publicly available wellness and stress management apps found only 2% had been evaluated in scientific studies^26^

Few self-harm prevention apps have been specifically developed for children and adolescents^25 27 28^ with only two being evaluated with young people under the age of 18. In a feasibility study involving 38 participants aged 16–25 years, the Imaginator app was found to be safe and acceptable with moderate reductions in self-harm frequency at 3 months.^29^ The other app, BlueIce, was evaluated in an open study with 40 young people aged 12–17 and was found to be safe and acceptable with postuse improvements in self-harm, mood and anxiety.^30 31^ Data on self-harm app acceptability for young adolescents are therefore limited and no qualitative evaluation has been undertaken within the context of a randomised controlled trial (RCT).

### Objective

The aim of this paper is to summarise postuser feedback on app acceptability, use and safety from a cohort of adolescents randomised to receive BlueIce.^32^

## Methods

### Study design

A mixed methods study undertaking a content analysis of postuse interviews.

### Participants

All participants met the RCT inclusion criteria of being aged 12–17 years, had self-harmed at least twice in the past 12 months and were receiving treatment from NHS Child and Adolescent Mental Health Services (CAMHS).^32^ Of the 85 participants randomised to receive BlueIce, 60 (70.6%) completed the postuse survey at 12 weeks. Participants were aged 15.6 (SD=1.41) years, predominantly female (55, 92%) and of British white ethnicity (52, 87%).

### Usual care plus BlueIce

All participants received mental health interventions from specialist CAMHS clinicians. Participants received a combination of mental health and/or risk assessments; psychological therapy delivered individually or in groups, face to face or digitally, to young people and/or their carers; pharmacological treatment; multidisciplinary team review and discussion; and liaison with other services and professionals. Interventions focused on their primary mental health problems and self-harm. In addition, participants were also provided with the BlueIce self-help app.

Participants, or if under 16, their carers, were contacted to confirm the young person’s telephone details and phone operating system. The young person was then sent a single-use download code to install BlueIce, a link to an informational video providing an overview of the app and a contact link in case of problems. Screenshots are available from the following link: https://apps.apple.com/us/app/blueice/id6468999790.

BlueIce can be accessed 24/7 and includes a (1) mood checker for emotional monitoring, (2) a mood lifter of ideas to improve mood and prevent self-harm and (3) automatic routing through safety checks to delay or prevent self-harm to emergency contact numbers. It is password protected, data are saved on the phone and no information is transmitted or stored elsewhere, or monitored by healthcare professionals. Versions are available for iPhone and Android-based phones. A detailed description of the app is provided elsewhere.^30 31^

### Postuse interview

A postuse interview, based on that used in an initial evaluation of BlueIce, was undertaken at 12 weeks.^31^ The interview was undertaken via telephone, by an experienced postgraduate researcher with individual participants. The researcher had no other role in the RCT. The interview consisted of 15 questions assessing four key areas. (1) App usage: whether BlueIce had been used, if so how often and if not, reasons why; whether BlueIce was used to monitor mood, when thinking about self-harm and if the young person had personalised the app and added their own content to the mood lifter. (2) Helpfulness: whether BlueIce had prevented any episodes of self-harm and which parts of the mood lifter were most helpful. (3) Safety: whether it was not possible to use BlueIce or whether it was used but did not prevent an act of self-harm and reasons why. (4) Acceptability: what was helpful and unhelpful about BlueIce, any problems or changes the young person would like to see. Response options were categorical with open responses invited for six questions informed by two previous qualitative evaluations of BlueIce.^31 33^ These probed reasons why the app was not used or perceived to be unhelpful and how it could be improved. In addition, respondents were asked to provide overall ratings on a 1–10 Likert scale for ease of use, helpfulness, recommendation to a friend and an overall star rating (out of 5).

### Baseline mental health symptomatology

#### Self-harm

The Risk-Taking and Self-harm Inventory for Adolescents (RTSHIA)^34^ is a self-report measure of self harm developed in the UK for use with adolescents (aged 11–19 years). The RTSHIA self-harm scale consists of 18 items assessing the presence and frequency of a range of intentional self-injuries (eg, cutting, burning, overdoses, suicide attempts) over the preceding 6 months. Previous research has found the RTSHIA to have good reliability and validity.^34^

In addition, respondents completed eight closed response questions from the Avon Longitudinal Study of Parents and Children (http://www.alspac.bris.ac.uk). These assessed the frequency, method, and reason for self-harming, suicidal intent and whether medical help was sought after the most recent incident.

#### Depression

The Mood and Feelings Questionnaire^35^ is a 33-item self-report questionnaire which assesses depressive symptoms over a 2-week period. Responses are rated on a 3-point scale: Not true (0), Sometimes true (1) and True (2). Total scores can range from 0 to 66, with a total score of 27 and above being associated with severe depression.^36^

#### Anxiety

The Revised Child Anxiety and Depression Scale^37^ is a 47-item self-report questionnaire assessing Diagnostic and Statistical Manual of Mental Disorders fourth edition (DSM-IV) criteria for social phobia, separation anxiety, obsessive compulsive disorder, panic disorder, generalised anxiety disorder and low mood. Each item is rated on a 4-point Likert scale of frequency ranging from Never (0) to Always (3). Items are then summed to produce subscale and total anxiety scores. There are age and gender-related norms for identifying clinically significant scores (total score ≥64–80).

### Analysis

This is a mixed methods study undertaking a content analysis of postuse interviews.^33^ As the interview responses were a mix of categorical data, Likert scale responses and brief open statements, content analysis was appropriate for simple reporting of common issues within the dataset.^38^ Individual telephone interviews were transcribed verbatim with 266 open comments being identified. An initial deductive approach, informed by previous qualitative evaluations of BlueIce,^31 39^ generated key categories for the coding framework. Two authors (PS and BC) independently reviewed and inductively coded all open comments and then met to discuss codes and finalise the coding framework. This was an iterative process where codes were revised, additional codes identified, the codebook checked for consistency and frequency counts obtained. The final coding frame can be found in the online supplemental material, with the key categories, codes and example quotes. All data were coded by both PS and BC, and Cohen’s kappa was run to determine intercoder reliability, which indicated strong agreement (K=0.87).

10.1136/bmjment-2023-300961.supp1 Supplementary data

Statistical analyses were undertaken using the Statistical Package for the Social Science (SPSS V.27.0. IBM 2020). Simple descriptives summarise demographic data, participant flow, symptomatology and postuse survey responses. Student’s t-tests explored differences between scores on standardised questionnaires, with non-parametric χ^2^ tests examining categorical data.

## Findings

### Survey respondents and non-respondents

A comparison was undertaken of the age, birth gender, ethnicity, self-harm frequency, prescribed mental health medication, number of mental health service contacts and baseline symptomatology of those who completed the postuse interview (n=60) and those who did not (n=25).

The summary in table 1 shows no statistically significant differences on any variable between interview responders and non-responders.

**Table 1:** Comparison between postuse survey respondents (n=60) and non-respondents (n=25)

Variable	Respondents (n=60)	Non-respondents (n=25)	P value
Age	15.57 (1.41)	15.55 (1.41)	0.970
Birth gender female	55 (91.67)	23 (92.0)	0.959
Ethnicity British white	52 (86.7)	23 (92.0)	0.487
Self-harmed 3 or more times in the past 4 weeks	37 (61.67)	18 (72.0)	0.364
Prescribed mental health medication in 6/12 before randomisation	24 (40.0%)	9 (36.0%)	0.730
Number of child mental health contacts in 6/12 before randomisation	8.0 (9.18)	9.76 (13.04)	0.481
**Self-harm**	**Mean (SD)**		
RTSHIA	20.10 (9.04)	22.08 (8.47)	0.339
**Mood**			
MFQ	45.10 (11.54)	48.12 (7.28)	0.230
**Anxiety**			
RCADS	80.88 (17.97)	83.64 (21.33)	0.544

MFQ, Mood and Feelings Questionnaire; RCADS, Revised Child Anxiety and Depression Scale; RTSHIA, Risk-Taking and Self-Harm Inventory for Adolescents.

### Baseline symptomatology

The primary mental health diagnoses of interview respondents were mixed mood/anxiety disorders (n=20, 33%), anxiety disorders (n=15, 25%), mood disorders (n=14, 23%), eating disorders (n=4, 6.7%), stress reactions (n=4, 6.7%) or other disorders (n=3, 5.0%). One-third (21, 35%) also presented with a diagnosed or suspected (referred for further assessment) neurodevelopmental disorder.

All participants had self-harmed in the 6 months before study enrolment with 46 (76.7%) self-harming within the past 2 weeks. Self-harm was frequent, with 47 (78.4%) self-harming more than five times in the past 6 months. 50 (83.3%) reported that they had seriously thought about killing themselves with 26 (43.3%) making at least one attempt to end their life.

### BlueIce feedback

#### App usage

57 (95%) respondents reported using BlueIce, with 44 (73.3%) using it six or more times over the past 12 weeks. Of the three who did not use BlueIce, one reported an improvement in their mental health, one forgot and one offered no explanation.

The mood diary was used by 54 (94.7%) of those who used the app. Positive benefits included: providing an overview of mood, ‘it helped me keep track of my mood’; externalising feelings, ‘it helped to get it out of my head’; and identifying patterns and triggers, ‘writing notes …. was quite nice because you could see what was making you sad’. A few negative comments related to remembering negative emotions, ‘writing down how I was feeling was just sort of reliving it’ or practical issues such as the ‘moods on it were limited’.

47 (82.4%) used BlueIce when thinking about self-harming. Reasons for not using the app were: forgetting (n=4), ‘it didn’t really come to my mind in those moments’; mental health improving (n=2), ‘my mental health has been a lot more positive over the past couple of months’; feeling too emotionally overwhelmed (n=2), ‘the urge was too strong’; a lack of motivation (n=1), ‘I couldn’t be bothered’; and a lack of belief in the app (n=1), ‘I don’t mean to be rude but I just didn’t think it would help’, with one being unable to identify a reason. 41 of the 47 who used BlueIce when distressed could recall the frequency of use with 24 (59%) using it between one and five times and 17 (41%) using it more than six times.

The mood lifter was personalised by 32 (56.1%) of those who used the app. Of the five sections that could be personalised, positive memories (good times, n=20) were personalised most often followed by social support (contact friends, n=18), distraction (do something, n=12), behavioural activation (get active, n=7) and uplifting music (listen and relax, n=4). Out of 10, the personalisation process was rated as very easy (x=8.72, SD=1.49, range 5–10).

#### Helpfulness

All sections of the mood lifter were used when thinking about self-harm. The most frequently used sections were distraction (do something, n=17), positive memories (good times, n=15) and social support (contacting friends, n=11). Three-quarters (36, 77%) of those who used BlueIce when thinking about self-harm reported that it prevented at least one episode of self-harm, with eight (17%) reporting that at least six episodes were prevented.

Not all self-harm urges were successfully prevented. 33/47 (70%) reported times they used the app but went on to self-harm, with 40/57 (70%) reporting occasions they had self-harmed but had not used the app. Reasons why the app was not helpful or used focused around three areas. First, a number reported feeling overwhelmed by strong emotions that prevented them focusing on the app; ‘I was really overwhelmed and couldn’t properly focus on anything’ or ‘it was when I was really stressed and then it feels like nothing would really help’. Second, a number reported that they had already convinced themselves that the app was not going to be helpful; ‘because my head was just like no this won’t help’ or ‘I just knew it wasn’t going to help’. Finally, a number described how they had already decided to self-harm; ‘I had already decided that was what I was going to do’ or ‘I was at that point really where it was going to happen’.

#### Safety

A total of 18 (31.6%) identified times they were unable to use BlueIce. Nine (50.0%) identified contextual factors such as ‘not allowed phones at school’, ‘not having access to my phone at night’ or ‘I don’t always have my phone on me’. Five (27.8%) identified phone-related issues such as a dead battery or changing their phone and not reinstalling the app. Two (11.1%) identified forgetfulness, ‘I just didn’t think about it’ with one (5.6%) identifying time as an issue, ‘…like you don’t always have time’. The remaining respondent identified her own personal dilemma over wanting to use the app but feeling that she did not deserve to be helped.

No young person identified any parts of the app which triggered any episodes of self-harm. Respondents reported not using various sections of the mood lifter but qualified this as a personal choice; ‘chill and relax weren’t particularly useful but that’s more of a personal preference than anything else’ or ‘I didn’t use contact a friend, not because they were unhelpful but because I get nervous’.

#### Acceptability

Overall, BlueIce was rated 4.09 (SD=0.75) out of 5 stars, with high mean ratings out of 10 for ease of use (8.70, SD=1.37) and good for acceptability (7.68, SD=2.05) and helpfulness (6.77, SD=1.72).

In terms of improvements, six suggested adding reminders to use the app, ‘something to help me remember in the moment would be perfect’ or for additional mood check notifications, ‘I was only given the option to add two notifications but if I’d had more I’d be able to use it more often’. Greater interaction with others was suggested by four, including talking ‘to other people using the app’, ‘to have a way to talk to my therapist on BlueIce’ or ‘others talking about their own experiences’. Two suggested amending or deleting entries in the mood diary, ‘edit previous days on the mood diary’. Personalisation of the app background, ‘like you could set a background with a nice photo or make your own colours’ or mood wheel, ‘make a mood board based on a few different things’ were suggested by two. More information on how to use the app was suggested by two, ‘if I had something that showed me how to use it that was actually within the app that would be good’. Others suggested improvements that BlueIce already had such as ‘adding more feelings to the mood wheel’ or ‘to be able to put pictures in’.

A summary of feedback is provided in table 2.

**Table 2:** Postuse feedback (n=60)

Item	n (%)
Did you use BlueIce over the past 12 weeks?	n=60
Did not use.	3 (5.0)
Used once or twice.	6 (10.0)
Used up to 5 times.	7 (11.7)
Used between 6 and12 times.	14 (23.3)
Used a couple of times each week.	8 (13.3)
Used more often.	22 (36.7)
Did you use the mood diary to record your mood?	n=57
Yes.	54 (94.7)
No.	3 (5.3)
Did you use BlueIce when you were distressed and thinking about self-harm?	n=57
Yes.	47 (82.4)
No.	10 (17.6)
How often did you use BlueIce when you were distressed and thinking about self-harm?	n=47
Once or twice.	14 (29.8)
Up to 5 times.	10 (21.3)
6–12 times.	12 (25.5)
Couple of times each week.	1 (2.1)
Used more often.	4 (8.5)
Could not remember.	6 (12.8)
What sections of the BlueIce mood lifter did you use?	n=47
Do something.	17
Good times.	15
Contact friends.	11
Listen and relax.	10
Ride it out.	9
Thinking traps.	8
Music library.	6
Get active.	5
Can not remember.	2
Did BlueIce prevent any episodes of self-harm?	n=47
No.	7 (14.9)
One or two episodes.	19 (40.4)
Up to 5 episodes.	9 (19.1)
6–12 episodes.	6 (12.8)
Prevented two episodes per week.	2 (4.3)
Could not remember.	4 (8.5)
Did you use BlueIce when you were thinking of self-harming and it did not help?	n=47
No.	12 (25.5)
Helped on some occasions but not all.	28 (59.6)
Used but always went on to self-harm.	5 (10.6)
No times it helped but no times it did not help.	2 (4.3)
Were there times you self-harmed and did not use BlueIce?	n=57
Yes.	40 (70.2)
No.	17 (29.8)
How easy (n=57) was it to use BlueIce on a scale of 1–10?	x=8.70 (SD=1.37), range 5–10
How helpful (n=57) did you find BlueIce on a scale of 1–10?	x=6.77 (SD=1.72), range 2–10
Would you (n=57) recommend BlueIce to others on a scale of 1–10?	x=7.68 (SD=2.05), range 2–10
How many stars (n=57) would you give BlueIce out of 5?	x=4.09 (SD=0.75), range 2–5

## Discussion

Postuse feedback is consistent with our previous open study which found that BlueIce was acceptable, used and safe.^31^ 82% used BlueIce when thinking about self-harm, with 77% reporting that it prevented at least one episode of self-harm. Nearly all (95%) used BlueIce at least once with 77% using it six or more times. In terms of content, all sections of the mood lifter were used and rated helpful, highlighting the importance of providing a toolbox of self-help strategies rather than focusing on one particular technique.

This evaluation has extended previous research by exploring reasons why BlueIce was not helpful or used. BlueIce did not prevent all episodes of self-harm. 70% of respondents reported occasions when they used BlueIce but still went on to self-harm. Similarly, 70% reported that they self-harmed without using the app. Reasons for BlueIce not helping or not being used were forgetting to use the app, feeling emotionally overwhelmed, a negative mindset or a prior decision to self-harm. These findings highlight how difficult it can be to engage in digital self-help at times of acute emotional distress.

Participants were also receiving face-to-face support from clinical staff, blind to arm allocation. Consequently, app use was not regularly reviewed or integrated within clinical care. The importance of ensuring that digital technologies are designed to support clinical services has been highlighted.^40^ Real-world implementation of digital technologies often fails because they are not used by patients or do not fit within clinical practice. Our findings show that although BlueIce was used there were a number of occasions when it was not. Similarly, although BlueIce was developed with mental health clinicians, the way it is integrated into clinical practice has not been determined. These findings suggest a hybrid model where app-delivered self-care is blended within face-to-face therapy.^41^ This would provide additional clinical support to encourage young people to maintain their positivity and to help them engage with mood-lifting activities at an earlier stage before their emotional distress becomes overwhelming. For clinicians, a brief app review at the start of each appointment would provide an overview of progress, identify helpful strategies and provide an opportunity to resolve any problems or issues.

BlueIce was acceptable to users, receiving an overall 4-star rating. This is consistent with findings from a review of 106 mental health apps where 69% were rated as 4 stars.^41^ The review found that characteristics such as ease of use, personalisation and range of content were particularly valued by users.^41^ These are core features of BlueIce. Ease of use was rated 8.7/10, over half (56%) personalised the mood lifter with all of the eight different options for managing distress and lifting mood being used by respondents.

### Strengths and limitations

These data are from a cohort of young adolescents aged 12–17 participating in an RCT. The cohort had persistent and repeated self-harm and significant mental health symptomatology and comorbidity. Although this study has a number of strengths, we acknowledge the following limitations. First, we relied on self-reports and there is no independent verification of app usage. The data we report may therefore be subject to recall or response bias where usage is under-reported or over-reported and content more favourably assessed. Second, participants were predominantly white British females, and the applicability of these findings to other ethnic groups or males cannot be assumed. Third, we were unable to interview 30% of BlueIce users. While we found no differences between respondents and non-respondents in terms of demographics, self-harm, mood or anxiety, we nonetheless cannot assume the generalisability of these findings.

### Clinical implications

We did not find any evidence that BlueIce was harmful or triggered any episodes of self-harm. Indeed, feedback was positive with self-reports indicating that BlueIce prevented a number of potential episodes of self-harm. These results support the acceptability of the BlueIce self-help app to young adolescents with severe mental health problems who repeatedly self-harm.

## Data Availability

Data are available upon reasonable request.
