# Supplementary material for: Acceptability, use and safety of the BlueIce self-harm prevention app: qualitative findings from the Beating Adolescent Self-Harm (BASH) randomised controlled trial
Source: BMJ Ment Health. 2024 Jun 25;27(1):e300961. doi: 10.1136/bmjment-2023-300961 (PMC11202734; doi:10.1136/bmjment-2023-300961)
Supplement: Supplementary data [file bmjment-2023-300961supp001.pdf]

Coding Frame V2

Category 1: Acceptability

Interview Question
Did you use the mood diary section to record your mood. If Yes, how often and was it helpful?

Category	Description	Codes	Example quotes
Mood Monitoring	Feedback about usage, helpfulness and specific benefits and limitations of the mood diary	1. Usage<br>- How often BlueIce was used.<br>- Factors determining use	"I used it sometimes, it kind of varied. Like it depended on the severity of my mood if I used it or not" (OX54)<br><br>"This was the bit I used the most. In January I used it almost every day" (OX109)
		2. Provides an overview of mood to help identify patterns and triggers.<br>- The benefits of reviewing diary entries over time<br>- The risks of this (remembering negative emotions)	"It was a good way of reflecting on my week and I I liked that you could make notes on your day, and I found that really helpful like seeing what was having a positive or negative impact on my mood" (OX64)<br><br>"if I'd had a prolonged streak of bad days though it was quite distressing to look back on" (OX116)
		3. Externalise feelings.<br>- The benefits of entering emotions in the mood diary	"you could write down what you were feeling. And yeah, it helped get it out of your head" (OX146)
		4. Facilitated self-help.<br>- Mood diary prompting self-help.	"When I put in that I was in a bad mood and then it gave me things to do, it helped me

1

			distract myself. (But) I found none of the things that were listed were interesting to me" (OX54)
		5. Unable to engage<br>- Unable to use mood monitoring because too distressed.<br>- Lacking motivation	"If I was in a bad mood or a rage or crying my eyes out then I was like "fuck an app" (OX54).<br><br>"But sometimes I'd go on the app and can't be bothered to type" (OX6)
		6. Practical issues<br>- Unable to delete entries.<br>- Moods too limited<br>- Routing through to calling someone is unhelpful	"I did find it helpful but I didn't like the fact that that you can't delete an entry. I would prefer to be able to delete it when you realise it's wrong" (OX157)<br><br>"The moods on it were limited so I'd add to the notes. There just weren't enough options so it didn't capture how I was feeling" (OX137)<br><br>"There could be more options after you say you're really upset and it takes you through to calling someone. It would usually end there and it doesn't feel helpful" (OX94)

**Interview Question**<br>Did you use BlueIce when you were distressed and thinking about harming yourself? (No)<br>Why were you unable to use BlueIce?			
**Category**	**Description**	**Codes**	**Example quotes**

2

Not used BlueIce	Did not use BlueIce when thinking about self-harm	1. Forgetting the app<br>- Unable to remember to use BlueIce<br>- Not motivated enough to use it	"I used it more after harming myself. In the moment it just didn't really cross my mind to use it" (OX78)<br><br>"I couldn't be bothered" (OX81)
		2. Urge to self-harm too strong.<br>- Feeling too emotionally overwhelmed to use BlueIce<br>- Not believing BlueIce would be helpful enough	"The urge was too strong" (OX136)<br><br>"I don't mean to be rude but I just didn't think it would help" (OX38)
		3. Mental Health improved.<br>- Did not think about self-harm so did not need to use the app.	"Well my mental health has actually been a lot more positive over the past couple of months cos I'm on medication and stuff like that, so I didn't actually feel like harming myself at all during this time. But when I was just feeling low it did definitely help. There were a few times when I felt quite down and I went into BlueIce and did some of the activities, they were very helpful as they were very distracting. It was then good like going back into mood diary and recording that after to see how my mood had changed, I found that really useful". (OX64)

Category 2: Helpfulness

**Interview Question**<br>Were there times you used BlueIce that it didn't help and you went on to harm yourself. (Yes)<br>Why do you think it didn't help?			
**Category**	**Description**	**Codes**	**Example quotes**
Used and self-harmed	Used BlueIce but it did not prevent an episode of self-harm	1. Emotions too strong for anything to help<br>- Overwhelmed by negative emotions.<br>- Decision to self-harm already made.	"If I am that low and there's so much going around in my head nothing really helps (OX33)<br>"I was too far down the mental health rabbit hole of this is what I'm going to do. I'd already made my mind up it was going to happen" (OX32)
		2. Content not helpful<br>- App content did not help	"It didn't really have something for me to focus on, like a game where you can sort of connect the dots...." (OX58)

**Interview Question**<br>Were there times when you self-harmed that you didn't use BlueIce (Yes)<br>What stopped you form using BlueIce?			
**Category**	**Description**	**Codes**	**Example quotes**
Not used and self-harmed	Reasons why BlueIce was not used before self-harm	1. Emotions too strong<br>- Didn't remember that the app was available to help<br>- Didn't feel like anything would help	"When you're in that situation and your head is kind of a mess you just don't think of it" (OX54)<br><br>"I wasn't necessarily like ooh let me go use the app, it just wasn't what I wanted to do cos I just wanted to hurt myself"(OX152)

		2. Phone unavailable.<br>- Didn't have access to phone.	"Just not having access to my phone" (OX119)

Category 3: Safety

Interview Question<br>Were there times you wanted to use BlueIce but couldn't (Yes)<br>Why were you unable to use it?			
**Category**	**Description**	**Codes**	**Example quotes**
Unable to use	Contextual, practical and personal reasons why unable to use BlueIce.	1. Phone not available<br>- Places or situations where the young person was unable to use BlueIce.<br>- Phone not working properly	"When I'm feeling proper distressed I don't always have my phone on me, like when I'm in school or when I'm in the shower. Also, I have this thing on my phone that my mum set up where apps shut down after a certain amount of time, like there's a screen time limit on them and BlueIce would shut down too." (OX94)<br><br>"The only times I self-harmed was when I didn't have my phone on me or my battery was dead or something." (OX86)
		2. Forgetfulness<br>- Forgot it was there.	"Only sometimes because I forgot" (OX4)
		3. Personal ambivalence<br>- Uncertain whether deserved help	"Sometimes there would be like a part of me that felt like I wanted to use it, but there was another part of me that said I shouldn't, because I felt like I shouldn't really get any help". (OX136)

Category 4: Modifications

**Interview Question**<br>Are there things we could add or changes that would make BlueIce more helpful?			
**Category**	**Description**	**Codes**	**Example quotes**
Changes	Suggestions for changes or additions to BlueIce	1. Add reminders.<br>- Additional reminders to use the app or rate mood.	"The only other thing I wrote down was about the reminders, they are really good but you have to remember to set them. Maybe it would be helpful to have automatic reminders, if you register with the app then something could ping you to say we're still here, or maybe throughout the day you could have little notifications come through with, like, quotes or something" (OX64).
		2. Personalise<br>- How the app could be personalised by users	"Maybe something you could personalise it with. Like you could set a background with a nice photo or make your own colours. Like chose the colour yourself" (OX36)
		3. App guide<br>- Information on how to use the app	"Not really, it was nothing problematic, I just think... I'd be like 'oh where do I find that again?'. If I had something that showed more how to use it that was actually within the app that would be good" (OX78).

		4. Edit diary entries.<br>- Editing or deleting mood diary entries.	"Just being able to edit previous days in the mood diary, just because sometimes I'll be staying up late and I'll be like oh I haven't done that today cos I like to try and fill it out as much as I can and then it gets just past midnight and it has to be for the next day" (OX169)
		5. Identify feelings.<br>- Additional questions to identify feelings.	"Maybe some like, when you're doing the mood diary like some buzz questions… like specific questions to help write down you're feeling. Like, are you feeling this? Cos like I said, verbalising it like can be difficult, so maybe it would be better to have a tick box or something, cos in the moment you don't really wanna try and speak about it, you just wanna agree with it if that makes sense?" (OX66)
		6. Communicating with others<br>- Messaging, communicating with professionals and other young people.	"Maybe like a section where you can talk to other people who are using the app, cos then you can both like help each other as well, cos 2 people using the app will have the same frame of mind and that's more helpful than talking to like… a normal person?" (OX105)
		7. Additional content<br>- Suggestions for additional content	"Yeah, like this other app calm harm, I feel like BlueIce could do some more of that because they are really similar apps but like, calm harm has things you can focus on, like with BlueIce where it suggests doing things, it sort of has things you can add to it like you can choose little mascots that correspond to the different things, and there's a breathing

			thing on there… which I'm not sure if BlueIce has, but there's lots of breathing things and I just find it quite helpful and I think could BlueIce could add some of those sorts of things to it"(OX146).
